# A very rare case of a subhepatic appendix of 20cm length in a girl

**DOI:** 10.11604/pamj.2023.44.12.38645

**Published:** 2023-01-06

**Authors:** Hind Cherrabi, Mohamed Amine Oukhouya

**Affiliations:** 1 Faculty of Medicine and Pharmacy of Agadir, Ibn Zohr University, Agadir, Morocco

**Keywords:** Sub-hepatic, very long appendix, anatomical variant, children

## Image in medicine

A 6-year-old girl was consulted for an appendicular syndrome that had been evolving for 3 days. The clinical examination revealed a febrile child with a temperature of 39°C and tachycardia. Abdominal examination revealed right hypochondrium defense and deep tenderness of the right flank and right iliac fossa. The biological work-up showed a predominantly neutrophilic hyperleukocytosis. Abdominal ultrasound revealed a subhepatic appendix 9mm in diameter associated with a well-limited hypoechoic collection in the right hypochondrium 4cm in length with no obvious stercolith. Surgical exploration revealed a sub-hepatic and subserosal appendix of 20cm in length, which is a very rare anatomical variant and underlines the need to verify the convergence of the 3 caecal bands to determine the exact site of the appendicular base. The risk is to leave an appendicular stump in place.

**Figure 1:**
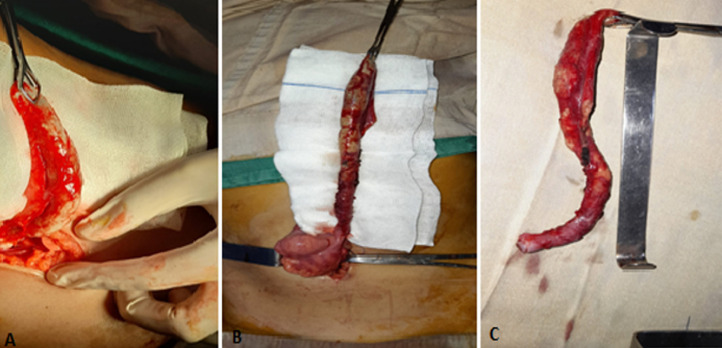
intraoperative images showing: A) the initial appearance of the appendix; B) extraction of the sub-hepatic appendix after retrograde dissection of the appendicular meso; C) surgical specimen of the appendix after appendectomy

